# Sertoli Cells Maintain Leydig Cell Number and Peritubular Myoid Cell Activity in the Adult Mouse Testis

**DOI:** 10.1371/journal.pone.0105687

**Published:** 2014-08-21

**Authors:** Diane Rebourcet, Peter J. O’Shaughnessy, Ana Monteiro, Laura Milne, Lyndsey Cruickshanks, Nathan Jeffrey, Florian Guillou, Tom C. Freeman, Rod T. Mitchell, Lee B. Smith

**Affiliations:** 1 MRC Centre for Reproductive Health, University of Edinburgh, The Queen’s Medical Research Institute, Edinburgh, United Kingdom; 2 Institute of Biodiversity, Animal Health and Comparative Medicine, University of Glasgow, Garscube Campus, Glasgow, United Kingdom; 3 Station de Physiologie de la Reproduction et des Comportements (PRC), Institut National de la Recherche Agronomique, UMR 6073 INRA-CNRS-Université de Tours, Nouzilly, France; 4 The Roslin Institute, University of Edinburgh, Easter Bush, Edinburgh, United Kingdom; University of Muenster, Germany

## Abstract

The Sertoli cells are critical regulators of testis differentiation and development. In the adult, however, their known function is restricted largely to maintenance of spermatogenesis. To determine whether the Sertoli cells regulate other aspects of adult testis biology we have used a novel transgenic mouse model in which Amh-Cre induces expression of the receptor for Diphtheria toxin (iDTR) specifically within Sertoli cells. This causes controlled, cell-specific and acute ablation of the Sertoli cell population in the adult animal following Diphtheria toxin injection. Results show that Sertoli cell ablation leads to rapid loss of all germ cell populations. In addition, adult Leydig cell numbers decline by 75% with the remaining cells concentrated around the rete and in the sub-capsular region. In the absence of Sertoli cells, peritubular myoid cell activity is reduced but the cells retain an ability to exclude immune cells from the seminiferous tubules. These data demonstrate that, in addition to support of spermatogenesis, Sertoli cells are required in the adult testis both for retention of the normal adult Leydig cell population and for support of normal peritubular myoid cell function. This has implications for our understanding of male reproductive disorders and wider androgen-related conditions affecting male health.

## Introduction

The Sertoli cells are essential regulators of testis differentiation and fetal masculinization through expression of SRY, secretion of AMH and induction of fetal Leydig cell development (reviewed in [1]–[4]). In the adult, the primary functions of the Sertoli cells are to provide a physical framework to support germ cell survival and development (reviewed in [5]) and an appropriate environment to ensure germ cell maturation [6], [7]. The Sertoli cells also act together with the peritubular myoid cells (PTMC) to lay down the basement membrane surrounding the tubules [8]. It is currently unknown, however, whether the Sertoli cells also act in the adult to regulate the number and activity of other testicular somatic cell types.

There is now increasing evidence to suggest that testosterone levels in adult and ageing men are important for maintaining wellbeing [9]–[14]. In men, testosterone is produced largely by the testicular Leydig cells and as men age Leydig cell numbers are reduced [15], [16] and testosterone production per cell is also reduced [17]. Establishing what controls Leydig cell maintenance and function is therefore critical to understanding adult male health and wellbeing. The population of Leydig cells that maintains adult levels of testosterone develops after birth largely under the control of luteinizing hormone (LH). In mice lacking LH or the LH-receptor (LHCGR) adult Leydig cell numbers are about 10% of normal and testosterone levels are essentially undetectable [18]–[20]. Recent studies from our lab have shown, however, that the Sertoli cells are also essential for adult Leydig cell development [21]. A role for the Sertoli cells in adult Leydig cell development has also been suggested by Hazra and colleagues [22] who have shown that precocious androgen receptor (AR) expression in the Sertoli cells has knock-on effects on the Leydig cells. Once established, however, the Leydig cell population is quite stable through most of adulthood and cell division is very rare under normal circumstances [23]–[25]. Even withdrawal of LH support, while reducing testosterone production markedly, has little effect on Leydig cell number [26], [27]. It is not clear, therefore, whether the Sertoli cells continue to play a role in regulating adult Leydig cell maintenance or whether the Leydig cells are largely autonomous, dependent only on hormonal regulation.

Use of controlled cell ablation to study development and function has a long history of utility in testis biology. Studies using cytotoxins such as busulfan (for germ cell ablation) [28], [29], and ethane dimethane sulphonate (EDS) (for Leydig cell ablation in rats) [30] have uncovered previously intractable aspects of testis function. Similar studies to examine the effects of Sertoli cell ablation on adult testis function have not been possible, however, as cytotoxins that specifically target the Sertoli cells have not been available. To address this need, we have developed a novel transgenic mouse model that permits controlled and specific ablation of Sertoli cells at any chosen age via Diptheria-toxin (DTX)–mediated induction of apoptosis [21]. In this study we show that specific and acute ablation of Sertoli cells in adulthood causes loss of all germ cells, apart from elongated spermatids, and a reduction in PTMC activity although the PTMC layer remains effective at excluding immune cells from the lumen of the seminiferous tubules. Most importantly, we also show for the first time that, in addition to their established role in supporting spermatogenesis, the Sertoli cells are critical for maintaining Leydig cell numbers in the adult testis. These findings significantly alter our understanding of adult testis function, with potential implications for male reproductive and general health.

## Materials and Methods

### Ethics Statement

Mice were housed and bred under standard conditions of care. Experiments passed local ethical review and were conducted with licenced permission under the UK Animal Scientific Procedures Act (1986), Home Office licence number PPL 60/4200.

### Breeding of transgenic mice, Genotyping

Mice were generated as previously described [21] using the Cre/Lox cell specific recombination system. Amh-Cre;iDTR, Stra8-Cre;iDTR and the CSFR-GFP^+/−^ (MacGreen) mice bred to Amh-Cre;iDTR mice were identified by genotyping from ear or tail DNA for the presence of *Cre* and *iDTR* using standard PCR with appropriate primers listed in Table S1. Inheritance of the GFP transgene was also verified in the MacGreen cross-bred animals. For the phenotypic study, matings were designed to generate only homozygous Amh-Cre^+/+^;iDTR^+/+^, Mac-Green and Stra8-Cre^+/+^;iDTR^+/+^. Adult animals (post-natal day 50: pnd50) were treated with a single dose of DTX (100 ng) as previously described [21].

### Tissue collection

**Figure 1:**
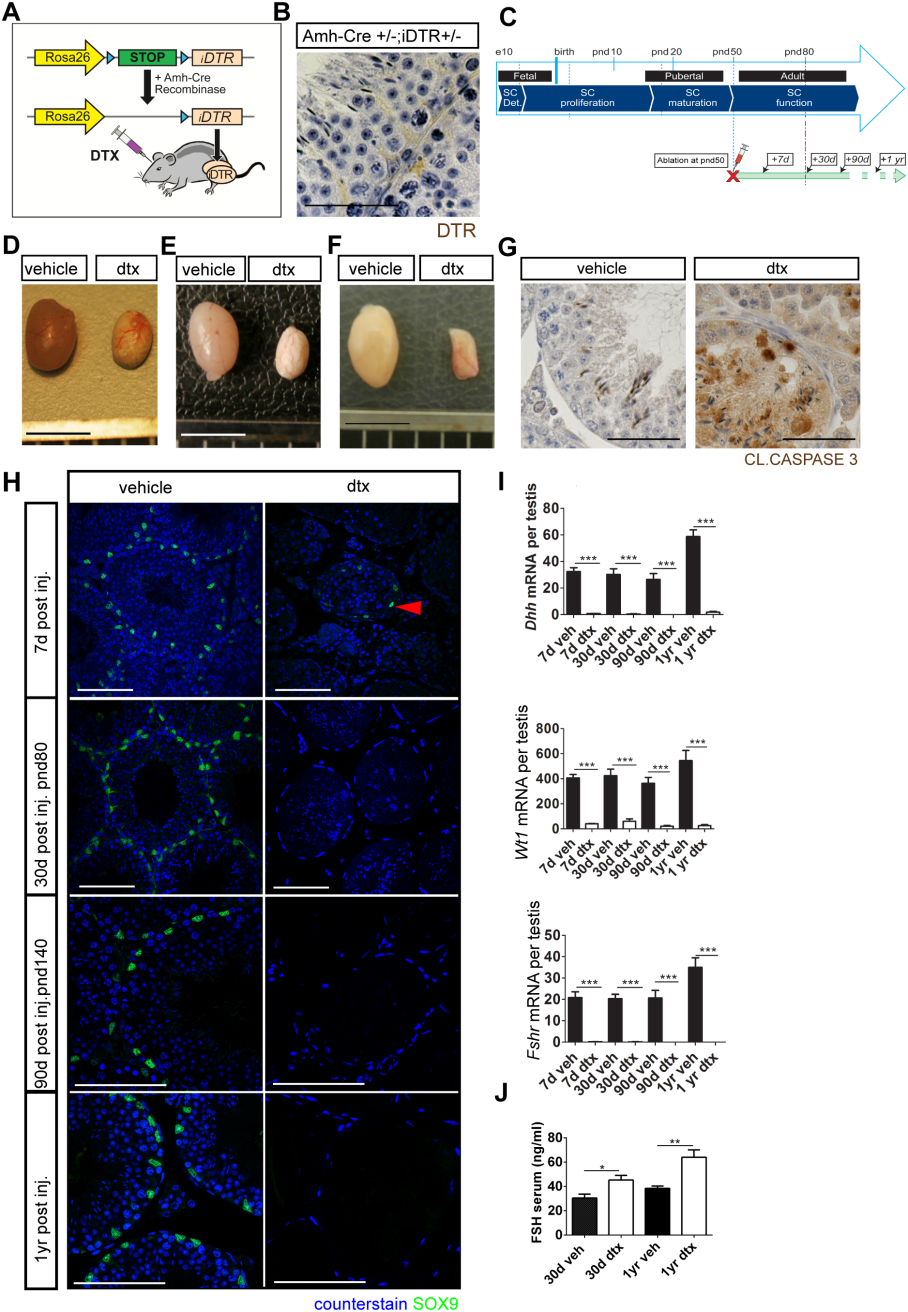
Sertoli cell-specific ablation in adulthood. (A) Amh-Cre;iDTR model. (B) DTR expression is restricted to Sertoli cells (scale bar: 50 µm). (C) Schematic representation of experiments in this study showing the time points of ablation relative to testis development. DTX-induced testicular atrophy (D) 7 days, (E) 30 days or (F) 1 year after injection in adulthood (scale bar: 500 m). (G) Immunolocalisation for CLEAVED CASPASE3 shows that apoptosis is restricted to Sertoli and germ cells (scale bar: 50 µm). (H) SOX9 expression is reduced but is still present in occasional tubules 7 days post-Sertoli cell ablation (arrowhead). However, SOX9 is completely absent 30 days post-Sertoli cell ablation (scale bar: 100 µm). (I) Sertoli cell ablation is mirrored by a decrease in Sertoli cell specific transcript expression: *Dhh*, *Fshr* and *Wt1* (one-way ANOVA n = 5–9, ***P<0.001). (J) Circulating FSH concentrations at d80 (one-way ANOVA n = 9–12, *P<0.05, **P<0.01). d,days; yr,year; dtx, injected with toxin; veh: vehicle control.

Animals were culled at different times after DTX injection (see Figure 1C) by CO_2_ asphyxiation and subsequent cervical dislocation. Blood was harvested by cardiac puncture for hormonal profile analysis. Body weight and reproductive organs (testis and seminal vesicle) weights were recorded. Collected tissues were either fixed or frozen for RNA or protein analysis.

### RNA isolation and real-time PCR

Testis RNA was extracted using Trizol (Life Technologies, Paisley, UK) and levels of specific mRNA species were measured by real-time PCR following reverse transcription of the isolated RNA. Total RNA was reverse transcribed at 50°C using random hexamers and Moloney murine leukaemia virus reverse transcriptase (Superscript III, Life Technologies) as described previously [31]. To allow specific mRNA levels to be expressed per testis and to control for RNA extraction efficiency, RNA degradation, and the reverse transcription step, 5 ng external standard (luciferase mRNA: Promega UK, Southampton, UK) was added to each testis at the start of the RNA extraction [32]. The real-time PCR approach used the SYBR green method in a 96-well plate format with a Stratagene MX3000 cycler. Reactions contained 5 µl 2xSYBR mastermix (Agilent Technologies, Wokingham, UK), primers (100 nM) and template in a total volume of 10 µl and a melting curve analysis was carried out on the products formed. All primers were designed by Primer Express 2.0 (Applied Biosystems, Warrington, UK) using parameters previously described [33] and the primers used are described in Table S1. Transcript levels were normalised relative to the luciferase external standard, which generates a value of transcript expression per testis [34].

### Immunostaining and Stereology and histology

Tissues were fixed in Bouins for 6 hours, stored in ethanol (70%) and embeded in paraffin or in resin (see below). Tissue sections (5 µm) were dewaxed in xylene then rehydrated in graded ethanol solutions. For histology, slides were stained with Hemaetoxylin/Eosin and Picrosirius Red using standard protocols. For immunostaining, slides were subjected to antigen-retrieval in a pressure cooker for 5 minutes with 0.01 M citrate buffer (pH 6.0). To quench endogenous peroxidases activity, slides were incubated in 0.3% hydrogen peroxide (v/v) in Tris-buffered saline (TBS) for 30 minutes at room temperature (RT). Nonspecific activity was blocked using the appropriate normal blocking serum (10% normal serum (vol/vol; Biosera) and 5% BSA (wt/vol; Sigma-Aldrich, Gillingham, UK) in TBS for 30 minutes at RT followed by incubation overnight at 4°C with the primary antibody diluted in blocking serum. The primary antibodies used in this study are detailed in Table S2. After washing, slides were incubated for 30 minutes at RT with the appropriate secondary antibody. For diaminobenzidine (DAB) visualisation, the secondary antibody was conjugated to biotin and diluted 1/500 in the blocking serum. This step was followed by incubation with horseradish peroxidase-labeled avidin-biotin complex (VectorLabs, Peterborough, UK) for 30 minutes at RT then development in DAB (Immpact DAB; VectorLabs, Peterborough, UK). Slides were counterstained with haematoxylin, dehydrated and mounted with Pertex mounting medium (Cell Path, Hemel Hempstead, UK). For immunofluorescence, the secondary antibody was conjugated to peroxidase and diluted 1/200 in blocking serum and left on the slides for 30 minutes at RT. Sections were then incubated with a fluorescein Tyramide Signal Amplification system (‘TSA', Perkin Elmer) for 10 minutes at RT according to the manufacturer’s instructions. Sections were counterstained in Sytox Green (Molecular Probes, life technologies, Paisley, UK) for 10 minutes at RT and mounted in PermaFluor mounting medium (Thermo Scientific, UK). Sections incubated with no primary antibody were used as negative controls. To ensure reproducibility, 4 to 6 different animals from each group were tested and sections from control and DTX-treated animals were processed simultaneously on the same slide.

For stereological analysis, testes were embedded in Technovit 7100 resin, cut into sections (20 µm), and stained with Harris’ hematoxylin. The total testis volume was estimated using the Cavalieri principle [35]. The optical disector technique [36] was used to count the number of Leydig cells in each testis. The cells were recognised by their position, round nucleus and relatively abundant cytoplasm as described previously [18], [37]–[39]. After DTX injection the morphology of some of the Leydig cells started to change (e.g. reduction in cytoplasmic volume) as described in Results. These cells were included in the Leydig cell count while they maintained morphological features consistent with Leydig cells. Immunohistochemical staining confirmed that only cells morphologically-recognisable as Leydig cells stained with Leydig cell markers (eg HSD3B) after DTX treatment. The numerical density of Leydig cells was estimated using an Olympus BX50 microscope fitted with a motorized stage (Prior Scientific Instruments, Cambridge, UK) and Stereologer software (Systems Planning Analysis, Alexandria, VA, USA). To illustrate testis histology semi-thin 2.5 µm sections were cut from tissues embedded in resin and stained with hematoxylin and eosin.

### Measurements of testosterone, LH and FSH

After blood collection, sera were separated and stored at −20°C. Serum testosterone was measured using a commercial kit (Demeditec Diagnostics, Germany) while FSH and LH were measured by ELISA as previously described [40]. The inter coefficient of variance (CV) was 12% for LH and FSH at 2.5 ng/ml and 1.2 ng/ml. The intra CV was <10% for LH and FSH at 0.1 ng/ml to 10 ng/ml. The inter CV was 10.9% for Testosterone at 2.5 ng/ml and 8.9% at 6.0 ng/ml. The intra CV was <8% for Testosterone at 0.1 ng/ml to 25 ng/ml.

### Tubule Permeability Study

Testes were collected from Amh-Cre;iDTR animals 7 days after treatment with DTX at pnd50. The interstitium of the testes was injected with 15 µl of 10 mg/ml EZ-Link Sulfo-NHS-LC-Biotin (Pierce, Rockford, IL) freshly diluted in PBS containing 0.01 M MgCl_2_ and the testes were left on ice for 30 min. The contralateral testis was injected with vehicle (0.01 M MgCl_2_ in PBS) as a control. After incubation, the testes were fixed in Bouins for 6 hours, embedded in paraffin and processed for immunohistochemistry as above.

### Figure Handling

Figures were compiled using Adobe Photoshop CS6 and Adobe Illustrator CS6 (Adobe System Inc., Mountain View, CA, USA).

### Statistical analysis

Data were analysed using Student T test, one-way ANOVA with the appropriate post hoc tests (Turkey’s multiple comparison or Dunnet’s tests), Bartlett’s test or Chi-square test. Specific details for each experiment are provided in figure legends. Graph Prism version 5 (GraphPad Software Inc., San Diego, CA, USA) or Minitab (Minitab Inc, State College, PA, USA) were used for analysis. When required, data were normalised by box-cox transformation. Values are expressed as means ± SEM.

## Results

### Sertoli cell specific ablation in Amh-Cre;iDTR mice

To determine the fundamental role of Sertoli cells in the adult testis we bred an Amh-Cre^+/−^ line [41] to mice carrying a Cre-inducible simian HBEGF (iDTR) [42] (Figure 1A), and confirmed that iDTR expression was restricted to Sertoli cells (Figure 1B). Pilot studies showed that injection of 100 ng DTX has no effect on the testes from Wild-type (WT), Amh-Cre^+/−^, or iDTR^+/−^ animals, but resulted in complete ablation of Sertoli cells in Amh-Cre^+/−^;iDTR^+/−^ mice (Figure S1), with no evidence of off-target effects in other organs (Figure S2). Following this demonstration Amh-Cre^+/+^;iDTR^+/+^ mice treated either with 100 ng DTX or vehicle were used for the remainder of the study to minimize animal numbers (Figure 1C). Treatment of adult pnd50) AMH-Cre;iDTR mice with 100 ng DTX led to a visible reduction in testis size when examined 7, 30 and 90 days post injection (Figure 1D–F). Histological and immunohistochemical examination of the testes showed Sertoli cell apoptosis and a striking reduction in Sertoli cell numbers (defined by immunolocalisation of SOX9) at 7 days post injection (Figure 1G, H). Measurement of the Sertoli cells markers *Dhh, Wt1* and *Fshr* by qPCR showed that expression was significantly reduced in the same period, with expression of all of these markers remaining low or absent up to one year after Sertoli cell ablation, (Figure 1I), consistent with complete loss of SOX9 immunolocalisation throughout this period (Figure 1H). Stereological analysis of the testes showed that less than 1% of the normal Sertoli cell population remained in the testis 30 days after DTX injection. Furthermore, circulating FSH concentrations were significantly increased both 30 days and one year post Sertoli cell ablation (consistent with removal of the Sertoli cell-dependent hypothalamic-pituitary-gonadal-axis negative feedback loop) (Figure 1J). Together these data confirmed successful acute ablation of the Sertoli cell population from the adult testis in the iDTR model.

### Ablation of Sertoli cells in adulthood induces loss of all germ cell-types, but does not impact on testicular architecture

**Figure 2:**
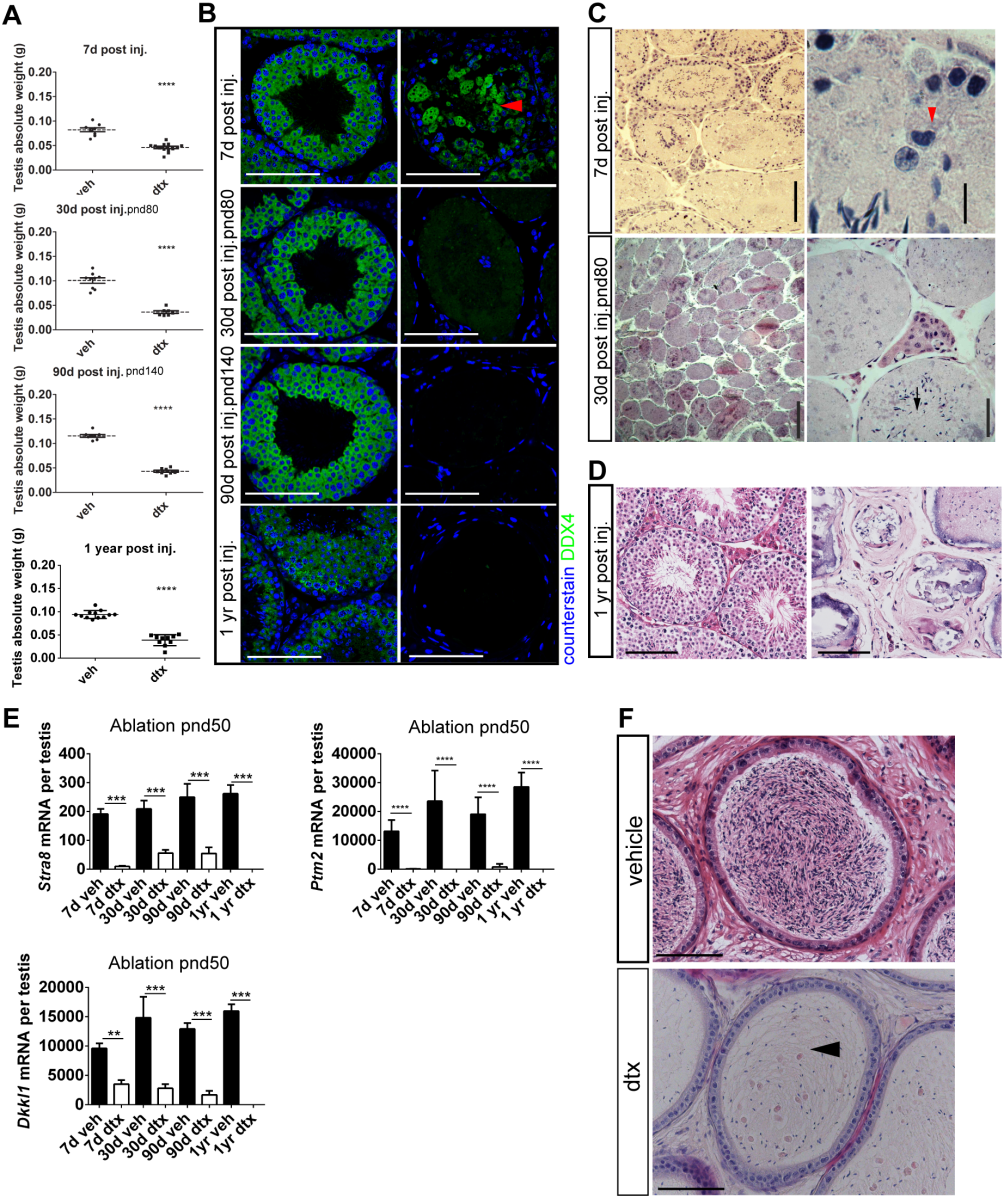
Germ cell loss after Sertoli cell ablation in adulthood does not impact testicular architecture. (A) Testis weight is decreased at all ages following Sertoli cell ablation in adulthood (T-test n = 8–12, ****P<0.0001). (B) Immunolocalization of DDX4 shows loss of Germ cells is complete 30 days following Sertoli cell ablation (scale bar: 100 µm). (C) Testicular histology (2.5 µm resin sections stained with hematoxylin and eosin) shows that intact tubules, containing spermatogonia, spermatocytes and spermatids, are present 7 days following Sertoli cell ablation although many of the spermatocytes (red arrowhead) are undergoing apoptosis. By 30 days the lumen of most tubules are either acellular or contain only elongated spermatids (black arrow). Low and high power pictures from treated animals are shown at each age. (D) Image on the right shows that one year after ablation tubular architecture is retained although the seminiferous tubules contain only calcium deposits and some elongated spermatids. The image on the left is a from a control, age-matched testis. (E) Expression of Germ cell markers *Stra8* and *Dkkl1* and *Ptm2* (one-way ANOVA n = 5–9, **P<0.05 ***P<0.001 ****P<0.0001). (F) Consistent with Germ cell loss, Cauda epididymides are devoid of spermatozoa and only cellular debris remains 30 days post Sertoli cell ablation (arrowhead) (scale bar: 100 µm). d,days; yr,year; dtx, injected with toxin; veh: vehicle control.

**Figure 3:**
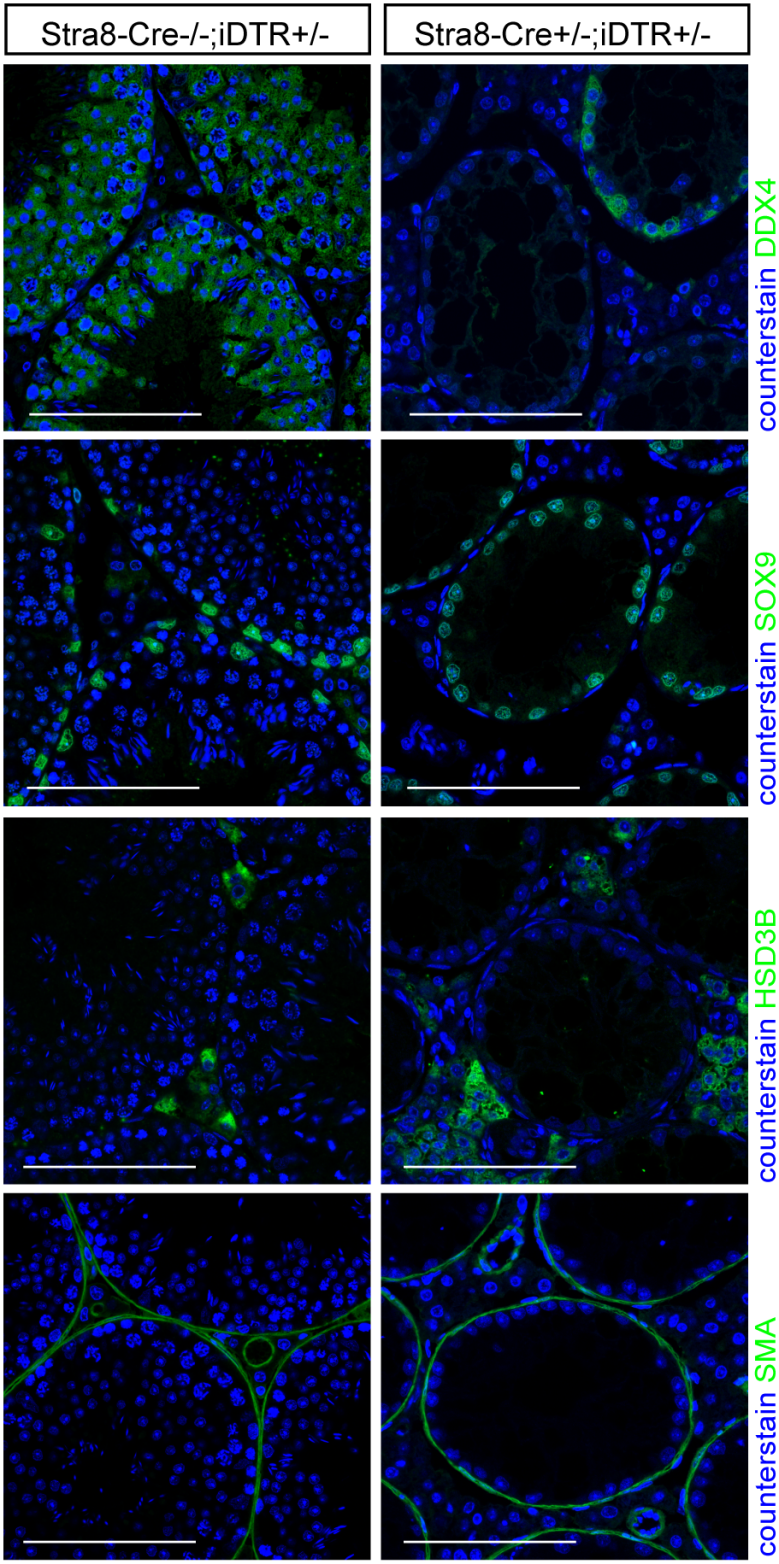
Germ cell-specific cell ablation in adulthood does not impact testicular architecture. Injection of DTX at pnd50 in Stra8-Cre^+/−^;DTR^+/−^ mice induces specific Germ cell ablation (DDX4 immunolocalization). Testis architecture is unaffected and key testicular somatic cells remain present - Sertoli cell (SOX9), PTMC (SMA), and Leydig cell (HSD3B) (scale bar: 100 µm).

Ablation of Sertoli cells at pnd50 led to a significant reduction in testis weight, 7, 30, 90 days and one year later (Figure 2A). This was primarily due to extensive loss of germ cells, which normally make up more than 80% of testicular cell types in the adult animal (Figure 2B). Seven days after DTX treatment the tubules remained intact and spermatogonia and spermatocytes were present although many of the spermatocytes and spermatids were undergoing apoptosis (Figure 2C). By 30 days after DTX treatment (pnd80) the lumen of most tubules was either acellular or contained only elongated spermatids (black arrow; Figure 2C). One year after DTX injection, the tubular structure of the testes remained morphologically intact although all of the tubules had shrunk and all contained calcium salt deposits. Elongated spermatids remained in some of the tubules, largely trapped within the calcium salts (Figure 2D). This loss of germ cells following Sertoli cell ablation was confirmed by loss of gene transcripts associated with specific germ cell stages (*Stra8* spermatogonia, *Dkkl1* spermatocyte, and *Ptm2* spermatid) (Figure 2E). Apoptotic loss of germ cells in situ was reflected in the significant reduction in numbers of spermatozoa present in the cauda epididymis 30 days after Sertoli cell ablation (Figure 2F). To exclude the possibility that germ cell loss would complicate our study of the somatic cell populations, we generated Stra8-Cre^+/−^; iDTR^+/−^ mice, which target iDTR expression specifically to the germ cell lineage (from pnd3) [43]. Injection of 100 ng of DTX at pnd50 induced cell-specific ablation of germ cells. Similar to mouse models of busulfan-mediated germ cell ablation [44], [45], we observed no gross somatic cell impact arising from germ cell loss (Figure 3) and Sertoli cell and Leydig cell numbers were not significantly different to control (data not shown).

### Sertoli cells regulate PTMC function in the adult testis

**Figure 4:**
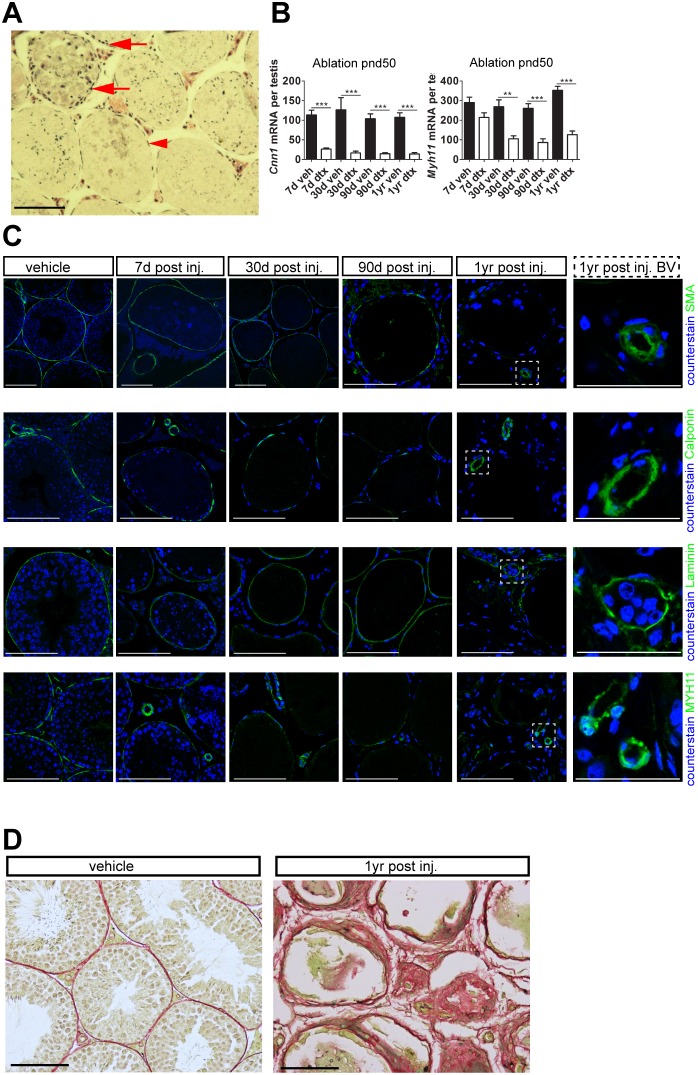
Sertoli Cells regulate PTMC function in adulthood. (A) Testicular histology (2.5 µm resin sections stained with hematoxylin and eosin) shows a single layer of PTMC is retained around seminiferous tubules (red arrowhead) 30 days after Sertoli cell ablation at pnd50 although some disruption of the PTMC layer can be noted (red arrows, associated with a small number of macrophage-infiltrated tubules) (scale bar: 100 µm). (B) Expression of PTMC markers *Cnn1* and *Myh11* (one-way ANOVA n = 5–9, **P<0.05, ***P<0.001). (C) SMA, Calponin (functional marker), laminin (basement membrane) and MYH11 expression is retained 90 days following Sertoli cell ablation at pnd50. Disruption of SMA, Calponin, laminin and MYH11 is observed one year following ablation (scale bar: 100 µm), although they remain unaffected in testicular vasculature (inset). (D) Consistent with loss of PTMC function a fibrotic phenotype (PSR staining) develops one year following Sertoli cell-ablation (scale bar: 100 µm). d,days; yr,year; dtx, injected with toxin; veh: vehicle control.

Following Sertoli cell ablation in the adult, a single PTMC layer, with a normal appearance, remained present around most tubules up to one year post ablation (arrowhead; Figure 4A). Levels of transcripts encoding the PTMC functional markers *Cnn1* and *Myh11* were significantly reduced, however, for up to one year after Sertoli cell ablation (Figure 4B). Consistent with this, immunohistochemical staining of Calponin and MYH11 was clearly reduced following Sertoli cell ablation (Figure 4C). However, the PTMC continued to express SMA and the localization of laminin appeared normal, suggesting retention of the basement membrane which maintains overall tubular structure in these animals (Figure 4C). Immunolocalization of all of these markers was unaffected in testicular blood vessels (Figure 4C). At one-year post Sertoli cell ablation there was loss of integrity in the connective tissue of the peritubular and interstitial region (Figure 4D), suggesting remodeling takes place over the longer-term. This re-modelling is likely to involve a number of cell types including the PTMC and interstitial cells. Together these results suggest that, while the presence of the basement membrane is sufficient to retain PTMC fate for several months after Sertoli cell ablation (as defined by SMA expression), the Sertoli cells are required in the adult animal to maintain normal PTMC function.

### The PTMC/basement membrane inhibits immune cell infiltration of seminiferous tubules

**Figure 5:**
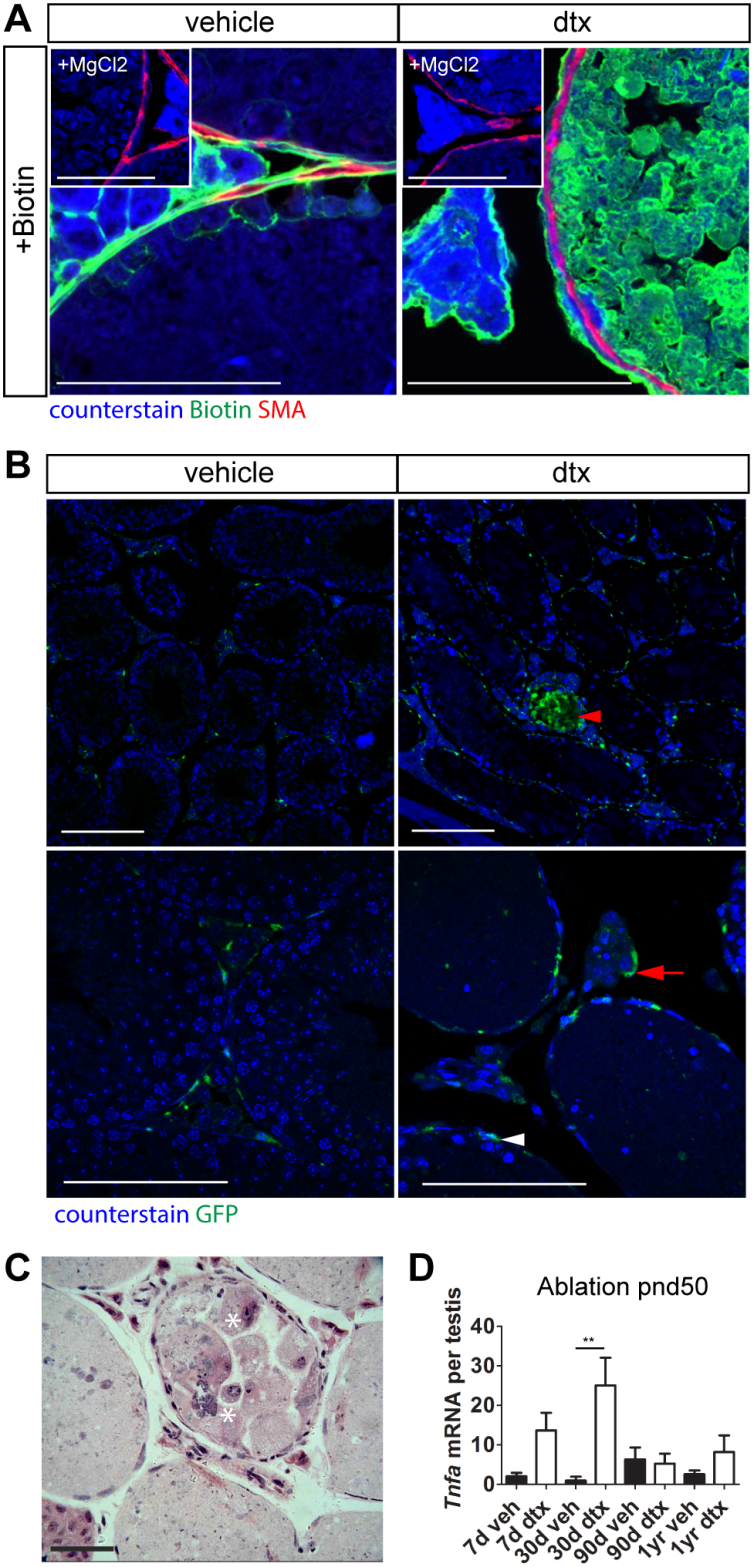
The PTMC/basement membrane inhibits immune cell infiltration of seminiferous tubules. (A) Biotin is prevented from entering the adluminal compartment by the Blood-Testis-Barrier in control animals. Conversely, 30 days post Sertoli cell ablation at pnd50, and despite the continued presence of the PTMC layer, biotin penetrates throughout the intratubular space. MgCl_2_: vehicle control. (B) Immunolocalization of macrophages (GFP) at 30 days following ablation at pnd50 using Amh-Cre+/+;iDTR+/+;CSFR-GFP mice (GFP antibody) shows that very few tubules are infiltrated by macrophages (red arrowhead). Macrophages are mainly present in the interstitium (arrow) or surrounding tubules (white arrowhead). (C) Sections from resinembeded testis highlighting the rare intra-tubular macrophage infiltration (asterisks). (D) Relative expression of the inflammatory cytokine *Tnfa* at key ages following Sertoli cell ablation at pnd50 (one-way ANOVA n = 5–9, **P<0.01) (scale bar: 100 µm). d,days; yr,year; dtx, injected with toxin; veh: vehicle control.

The blood-testis barrier (BTB) acts to regulate movement of substances into the seminiferous tubules and to prevent immune cell invasion of the tubules. In adult (pnd80) mice with Sertoli cell ablation, the PTMC layer remained intact in most tubules, which allowed us to determine the contribution of the PTMC and basement membrane to the BTB. Following biotin injection [46], the PTMCs created no barrier to biotin infiltration of the seminiferous tubules in the absence of Sertoli cells, consistent with the essential role of Sertoli cells in controlling the BTB (Figure 5A). To examine the possible function of the PTMC/basement membrane in exclusion of immune cells from the tubules, we bred Amh-Cre^+/+^;iDTR^+/+^ mice with CSFR-GFP (MacGreen) mice [47] to generate Amh-Cre^+/−^;iDTR^+/−^;CSFR-GFP^+/−^ mice. This permitted tracking of macrophages, following Sertoli cell ablation, through macrophage expression of GFP. Analysis confirmed that macrophage localization was largely restricted to peritubular and interstitial spaces with penetration of less than 1% of the total tubular sections observed despite the presence of cellular debris in all tubules (Figure 5B). This suggests that the PTMC plays a significant role in regulating immune cell access to the seminiferous epithelium. It is of interest that where macrophage invasion of the tubule did occur (Figure 5C asterisks), PTMC morphology was disrupted (Figure 4A, arrows), although it is not clear whether this was a cause or consequence of the macrophage invasion. To complete the kinetic analysis of immune cell response we determined relative expression of the inflammatory cytokine *Tnfa* [48] at each age. *Tnfa* expression was maximal 30 days post Sertoli cell ablation and fell to baseline levels by 90 days post ablation, consistent with resolution of the immune response (Figure 5D).

### Sertoli cell ablation in the adult mouse leads to a marked reduction in Leydig cell number

**Figure 6:**
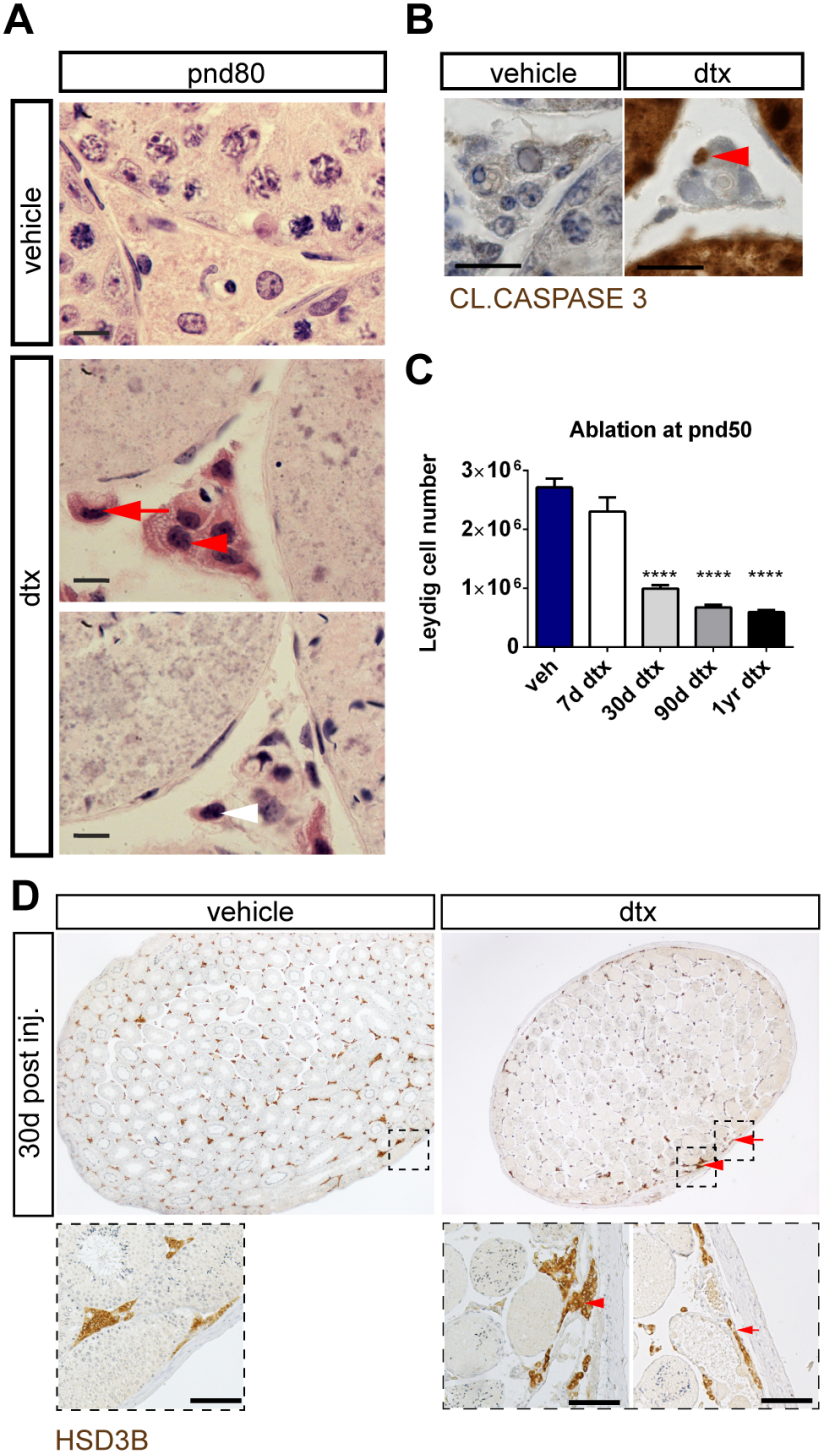
Sertoli cell ablation in adulthood induces apoptotic loss of Leydig cells. (A) Testicular histology on 2.5 µm resin sections (stained with hematoxylin and eosin) shows altered Leydig cell morphology 30 days after Sertoli cell ablation at pnd50. Leydig cells show increased vacuolization of the cytoplasm (red arrowhead), while some cells have an altered nuclear shape (red arrow) or reduced cytoplasmic volume after Sertoli cell ablation (white arrowhead). (B) Immunolocalisation for CLEAVED CASPASE3 shows Leydig cells undergo apoptosis (red arrowhead) (scale bar: 25 µm). (C) Total Leydig cell number per testis at key time-points following Sertoli cell ablation at pnd50 (one-way ANOVA n = 3–13, ****P<0.0001). (D) Leydig cells (HSD3B) are restricted to the rete (red arrowhead) and subcapsular region (red arrow) following Sertoli cell ablation at pnd50. (scale bar: 100 µm). d,days; yr,year; dtx, injected with toxin; veh: vehicle control.

Abundant Leydig cells remained in the interstitial tissue 7 days after DTX injection and the cells appeared normal. By 30 days after injection Leydig cells remained present singly or in groups throughout the interstitium but the nuclei of these cells often had an altered, elongated shape and the cytoplasm was vacuolated and/or reduced in size (Figure 6A), which suggested they may be undergoing apoptosis. This was confirmed through immunohistochemical localization of Cleaved Caspase 3 (Figure 6B). Measurement of Leydig cell numbers using the optical disector method showed that there was no change in cell number 7 days after Sertoli cell ablation but, by 30 days, Leydig cell number had reduced significantly to 37% of control and to 25% of control by 90 days post-ablation. There was no further change in Leydig cell number up to one-year post Sertoli cell ablation (Figure 6C) suggesting a stable threshold had been reached. Immunohistochemistry confirmed that Leydig cells were largely lost from the interstitial tissue and were only retained in the subcapsular region and around the rete testis (Figure 6D). Those Leydig cells that remained around the rete and subcapsular regions appeared to be histologically normal when observed by light microscopy. At 90 or 365 days after DTX injection, Leydig cell distribution was similar to that after 30 days although cells were more sparse in the interstitium. Together these data show that Sertoli cells are fundamentally required for retention of the Leydig cell population in the adult testis.

### Basal circulating testosterone is maintained but peak levels are lost after Sertoli cell ablation

**Figure 7:**
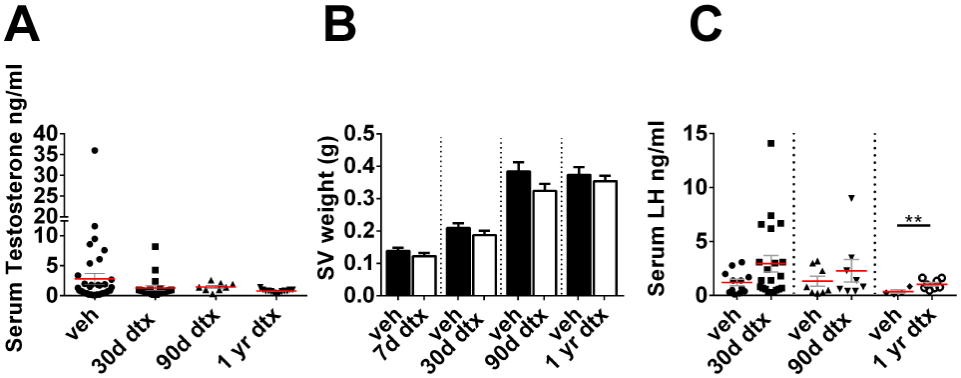
Adult Leydig Cell functionally compensate following Sertoli cell ablation in adulthood. (A) Serum testosterone (one-way ANOVA n = 7–14) (B) Seminal Vesicle weight (T-test n = 8–12) and (C) Luteinizing Hormone concentrations (T-test n =  n = 7–14, **P<0.01) in adulthood following Sertoli cell ablation at different ages. d,days; yr,year; dtx, injected with toxin; veh: vehicle control; SV:seminal vesicle.

**Figure 8:**
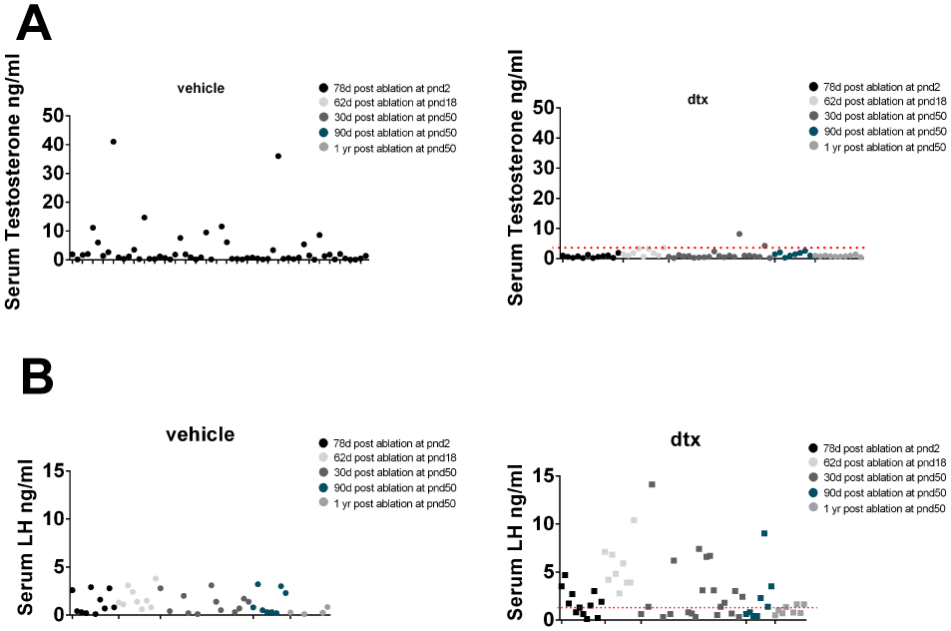
Changes in hormone pulsatility following SC ablation. Individual values for serum testosterone in vehicle-treated (A) and DTX-treated (C) animals. Individual values for serum LH in vehicle treated (B) and DTX treated (D) animals. Red dotted line indicates the mean of control animals. d,days; yr,year; dtx, injected with toxin; veh: vehicle control.

Despite the significant reduction in Leydig cell number, average circulating testosterone concentrations were not significantly different from control animals at any time-point following Sertoli cell ablation (Figure 7A), and seminal vesicle weights (Figure 7B) (a biomarker of circulating testosterone) did not differ from controls. Circulating testosterone levels in mice are pulsatile, however [49], and so there is often huge variation between animals depending on whether or not they were killed (and the blood sample collected) during a pulse. It is clear from Figure 7A that the variance in testosterone levels is greater in the vehicle controls than in the Sertoli cell-ablated animals (P<0.001 by Bartlett’s test) which suggests that there is attenuation of the testosterone pulses after Sertoli cell ablation. To examine this is more detail, data from all control animals (n = 64) and all Sertoli cell-ablated animals (n = 65), including data from animals with prepubertal Sertoli cell ablation [21], is shown in Figure 8. In control animals, two populations are apparent, animals with a basal level of testosterone and animals with an elevated level measured at some point during a testosterone pulse. It can also be seen, however, that the high “pulse” levels of testosterone are largely absent in the Sertoli cell-ablated animals (this difference is significant (P<0.008) by Chi-square testing). Mean LH levels were higher at all times after Sertoli cell ablation although this difference was only significant at one year (Figure 7C). As with testosterone, however, circulating LH levels in the mouse are pulsatile and when data from all animals is examined individually (Figure 8) it is clear that pulse levels of LH are higher after Sertoli cell ablation (P = 0.008, Chi-Square testing).

## Discussion

The adult Leydig cells differentiate from progenitor cells in the peritubular region of the testis with a possible contribution also coming from perivascular cells [50]. In the mouse this process starts between seven and ten days after birth [51] and, while the initial differentiation appears to be gonadotrophin-independent, development of a normal, functional adult Leydig cell population is critically dependent on LH [18]–[20], [52], [53]. The whole process is also dependent on the presence of the Sertoli cells; ablation of Sertoli cells in the neonatal period leads to a >90% loss of adult Leydig cells with mature cells only forming near the rete testis where Sertoli-like cells remain [21]. Once formed, activity of the adult Leydig cells is dependent on LH and osteocalcin and in the absence of either hormone Leydig cell activity is markedly reduced although cell numbers are largely maintained [27], [54]. Results reported here now show for the first time that the Sertoli cells are essential for maintenance of the adult Leydig cell population. The Sertoli cells, therefore, are critical regulators of the two major functions of the adult testis - germ cell development and androgen secretion.

There is no direct contact between Sertoli cells and Leydig cells and loss of adult Leydig cells following Sertoli cell ablation would suggest that there must be a Sertoli cell-derived factor(s) that is required to maintain the Leydig cells. This factor may act directly on the Leydig cell or may be mediated through the PTMC, which have been previously shown to regulate Leydig cell function [55]. The Sertoli cells are essential for maintenance of the PTMC phenotype in neonatal mice and loss of the PTMC is associated with loss of adult Leydig cell precursor cells and failure of adult Leydig cell development [21]. In the adult testis the PTMC remain intact after Sertoli cell ablation but there is a clear change in activity, which may at least contribute to loss of the adult Leydig cell population. The nature of the factor or factors derived from the Sertoli cells (and, possibly, the PTMC) and acting on Leydig cell maintenance is unclear at present. Previous studies have identified the presence of FSH-dependent Sertoli cell factors that can regulate Leydig cell activity [56]–[59], although their nature is also unknown and it is unclear whether they can act to maintain adult Leydig cell numbers. Earlier studies have also shown, however, that adult Leydig cell number is reduced in adult mice lacking FSH-receptors [60], which would be consistent with a link between these FSH-dependent factors and results reported here.

The maintenance of basal circulating testosterone, despite loss of 75% of the Leydig cell population following Sertoli cell ablation, suggests that the remaining Leydig cells may have become hyperactive. This effect is also seen following neonatal Sertoli cell ablation [21] and may be a result of altered LH pulse amplitude or may indicate that Sertoli cells normally act to constrain Leydig cell activity. A number of studies, mostly using *in vitro* systems, have reported the presence of Sertoli cell–derived factors, which inhibit Leydig cell function, although their nature is unknown [61]–[63]. It should be noted that the Sertoli cells have also been postulated to secrete factors which stimulate Leydig cell function such as DHH [64] and so effects of Sertoli cell ablation on remaining Leydig cells would be a balance between loss of stimulatory and inhibitory factors. Despite maintenance of basal testosterone levels, the Leydig cells were unable to generate testosterone pulses and so may be working at close to maximum activity. Loss of testosterone pulses had no effect on SV weight but consequences for other aspects of androgen-dependent growth and function remain uncertain at this point. Levels of FSH were doubled one year after Sertoli cell ablation which is a similar increase to that seen in castrate animals [65], [66]. This is likely to reflect loss of feedback control from the Sertoli cells (inhibins, follistatin) and loss of testosterone pulses.

Previous studies have shown that the adult Leydig cell population will regenerate from stem cells following ablation by the cytotoxic drug EDS [67], [68]. The question then arises why Leydig cells do not appear to regenerate following loss in this model of Sertoli cell ablation. One likely explanation is that regeneration requires the presence of the Sertoli cells, which our previous studies have shown to be essential for normal development of the adult Leydig cell population [21]. It has also been shown that Leydig cell regeneration following EDS is associated with high LH and inhibition of LH levels prevents cell differentiation [69]. Since LH levels do not rise markedly after Sertoli cell ablation this may also contribute to failure of Leydig cell regeneration. Interestingly, loss of Leydig cells from the testis following Sertoli cell ablation was spatially localized, with remaining cells mainly located adjacent to the rete testis or in the subcapsular region (Figure 7). The maintenance of Leydig cells around the rete testis following Sertoli cell ablation would be consistent with the Sertoli cell-like activity of the rete epithelial cells seen in neonatal mice [21] and suggests that these cells may retain a degree of Sertoli cell functional activity beyond puberty. It is less clear why Leydig cells are also retained or re-localize to the sub-capsular region of the testis following Sertoli cell ablation. It has been reported that mice with a gain of function mutation in the LH receptor gene show Leydig cell hyperplasia most frequently in the capsular region [70]. This would support the hypothesis that these subcapsular cells are different to the parenchymal Leydig cells either because of their localization or because they are a separate population of adult Leydig cells [71]. Either way, these subcapsular Leydig cells do not require Sertoli cell support and, if localization is the major influence, the presence of larger blood vessels in this area may be a contributory factor either through interaction with the vascular smooth muscle cells or through an altered growth factor environment.

As might be expected, Sertoli cell ablation in adult animals led to a rapid immune response in the testis with an increase in interstitial macrophages apparent by seven days and an increase in cytokine levels, which peaked at 30 days post-ablation. It is known that the resident testicular interstitial macrophages form a close physical and functional relationship with the Leydig cells [72], [73] and so changes in Leydig cell number/function following Sertoli cell ablation could also be related to changes in the macrophage population. It is unlikely, however, that this is a major contributing factor on its own to the marked loss of Leydig cells following Sertoli cell ablation as increased testicular macrophage numbers in other situations (e.g. orchitis [74], BTB disruption [75]) do not lead to marked changes in the adult Leydig cell population. Cytokines can increase or decrease Leydig cell function [76], which may contribute to changes in Leydig cell activity after Sertoli cell ablation but there is no evidence that they can induce Leydig cell apoptosis. Finally, the immune response in the testis is largely finished by 90 days after Sertoli cell-ablation in the adult but this is not associated with any marked change in Leydig cell function compared to 30 days.

The BTB is generated largely by the presence of specialised junctions between adjacent Sertoli cells near to the basement membrane [77] and it acts to restrict movement into the seminiferous tubules and to allow the Sertoli cells to regulate the internal environment of the tubules. The BTB develops in rodents between pnd15 and pnd19 [78]–[80] and formation of the barrier is an essential step in the development of spermatogenesis, any delay preventing the onset of meiosis [78], [81]. The contribution that the PTMC makes to the BTB is probably species dependent and early studies showed that in rodents the PTMC would act as a partial barrier to the penetration of lanthanum to the tubular epithelium [82], [83]. Studies reported here, however, show that in the absence of the Sertoli cells the PTMC fail to act as a barrier to the larger substance biotin. This would suggest that one function of the Sertoli cell is to maintain the barrier properties of the PTMC in the mouse. It is also clear, however, that even in the absence of the Sertoli cell the PTMC and underlying ECM is sufficient to exclude macrophages in most tubules. This answers a long-standing question in testis immunology, indicating that the PTMC is likely to contribute to the immune privilege of the seminiferous tubules. The mouse testis contains about 14 seminiferous tubules [84] and so the low level of macrophage invasion seen in testis sections suggests that, where infiltration occurs, it is a localized event and does not occur along the length of the tubule.

In a previous study a gradual loss of Sertoli cells was shown over a one-year period in mice lacking BCL2L2 (BCLW) [85]. This was associated with an initial hyperplasia of the adult Leydig cells lasting until 8 months followed by Leydig cell apoptosis so that by one year of age few Leydig cells remained in the testis [85]. What has not been clear in these studies was whether loss of Leydig cells was associated directly with loss of the Sertoli cells or was due to lack of BCL2L2 expression in the Leydig cells or another cell type. Results from our current study now show that loss of Leydig cells in BCL2L2-null animals is probably due, at least partly, to loss of the Sertoli cell population. The phase of Leydig cell hyperplasia seen in the BCL2L2-null mice did not, however, occur in the Sertoli cell-ablation model used here and may, therefore, have been a result of changes in Sertoli cell activity in the BCL2L2-null mice prior to cell death. Alternatively, the prolonged period of Sertoli cell death in BCL2L2-null mice may have been associated with increased LH levels, which counteracted the initial loss of Sertoli cell trophic factors to cause Leydig cell proliferation. Recent studies have shown that in mice lacking Dicer in the Sertoli cells there is abnormal development of the Sertoli cells followed by cell loss over a 6-month period [86], [87]. Following Sertoli cell apoptosis in these animals the testes contained abundant Leydig cells [87] but Leydig cell number was not measured and so it is not clear whether there was an overall change in Leydig cell number.

Data from this study should be read in context with previous results from our group showing developmental changes in the testis following Sertoli cell ablation in the fetus or in neonatal/prepubertal life [21]. The two studies have now examined different aspects of Sertoli cell function, their role in development of the testis and their role in maintenance of adult testicular function. The developmental studies have shown that during fetal and neonatal life the Sertoli cells are essential for the retention of tubular structure and for maintenance of PTMC phenotype [21]. As the animal ages into puberty [21] or adulthood (this study), however, the Sertoli cells are no longer required to maintain tubular structure or PTMC differentiated function, presumably because the basement membrane has developed to the point where it can support these functions. Both this study and the earlier developmental study [21] have shown that the Sertoli cells are essential at all ages for the maintenance of all germ cell populations, including spermatogonia. This may be due to maintenance of the specialized environment of the tubule lumen and is consistent with the absolute requirement for the Sertoli cells in order to retain a solute barrier (described here). The relationship between the Sertoli cells and the adult Leydig cells can also be traced through both studies. At puberty, differentiation of the adult Leydig cell population requires the presence of the Sertoli cells [21] and, once formed, these Leydig cells continue to require the Sertoli cells in order to prevent degeneration (this study). Therefore, while the function and role of the Sertoli cells changes during development and in adulthood, the cells remain critical at all time for normal testicular morphology, structure and activity.

The Sertoli cells are recognized as the primary regulators of testis development [2] and this study now shows that they continue to regulate the two major functions of the testis, germ cell development and androgen production, into adulthood. As such, therefore, this study represents a significant change in our understanding of events underpinning life-long support of testis function. In addition, the suggestion that trophic support from the Sertoli cell acts to maintain adult Leydig cell number and function may be an important factor in understanding life-long health in the male. In the human, numbers of Sertoli cells fall as men age [7] and ageing is also associated with a reduction in both number [15], [16] and function [17] of the adult Leydig cell population. In addition, while the concept of an ‘andropause’ remains contentious, the evidence linking (lower) testosterone levels to risk of cardiovascular disease [1], diabetes, obesity and metabolic syndrome [2]–[5], has become firmly established in recent years, and low testosterone is a predictor of early death [6]. The data from this study now suggests that the reduction in Sertoli cell number may be a major contributory cause of Leydig cell loss in the ageing testis. This highlights the importance of the Sertoli cells as both a potential target for endocrine disrupting chemicals or lifestyle influences (e.g. obesity) which could impact on Leydig cell function, and also as a novel target for potential therapeutics aimed at maintaining normal androgen profiles during ageing. Since the Sertoli cells are also under critical regulation by FSH and by androgen, long-term changes in the levels of these hormones may also contribute to more widespread effects on testicular function and health through changes in Sertoli cell activity.

## Supporting Information

Figure S1 **Diphtheria toxin-mediated Sertoli cell-specific ablation.** (A) Inheritance of iDTR and Cre Recombinase transgenes relative to testicular histology following DTX injection. Inheritance of both transgenes is required for Sertoli cell-specific ablation. 697 bp = iDTR-negative; 253 bp = iDTR-positive; 320 bp = Cre-negative (positive control for PCR amplification); 110 bp = Cre-positive; Marker (m); Water (w). (scale bar: 100 µm).
(TIF)

Figure S2 **Diphtheria-toxin does not cause increased apoptosis in other organs.** (A) Histology of non-gonadal tissues (Heart Brain Adrenal Kidney Liver and Spleen) demonstrating no off-target effects 7 or 30 days post-DTX injection (scale bar: 200 µm). (B) Immunostaining for CLEAVED CASPASE 3 protein in adrenal and kidney of adult Amh-Cre;iDTR vehicle or DTX-treated mice 7 days after ablation (scale bar: 100 µm). DTX treatment to ablate Sertoli cells engenders no off-target apoptosis in other tissues. d,days; dtx, injected with toxin; veh: vehicle control.
(TIF)

Table S1 **Primers used for Genotyping and qRT-PCR.**
(DOCX)

Table S2 **Details of Antibodies and detection methods used.**
(DOCX)
