# Supplementary figures and images for: Sertoli Cells Maintain Leydig Cell Number and Peritubular Myoid Cell Activity in the Adult Mouse Testis
Source: PLoS One. 2014 Aug 21;9(8):e105687. doi: 10.1371/journal.pone.0105687 (PMC4140823; doi:10.1371/journal.pone.0105687)

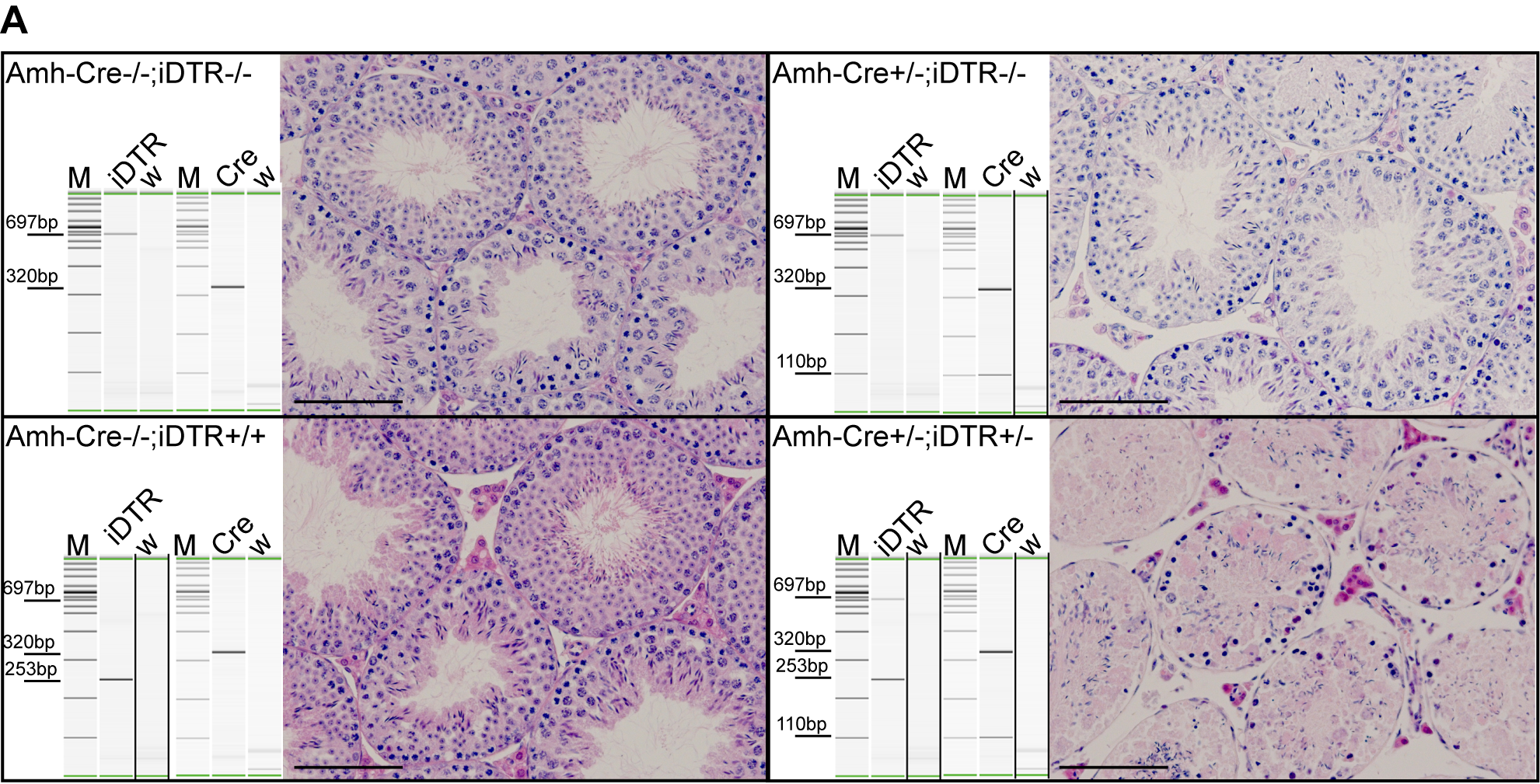

Supplement: Figure S1 — Diphtheria toxin-mediated Sertoli cell-specific ablation. (A) Inheritance of iDTR and Cre Recombinase transgenes relative to testicular histology following DTX injection. Inheritance of both transgenes is required for Sertoli cell-specific ablation. 697 bp = iDTR-negative; 253 bp = iDTR-positive; 320 bp = Cre-negative (positive control for PCR amplification); 110 bp = Cre-positive; Marker (m); Water (w). (scale bar: 100 µm). (TIF) [file pone.0105687.s001.tif]

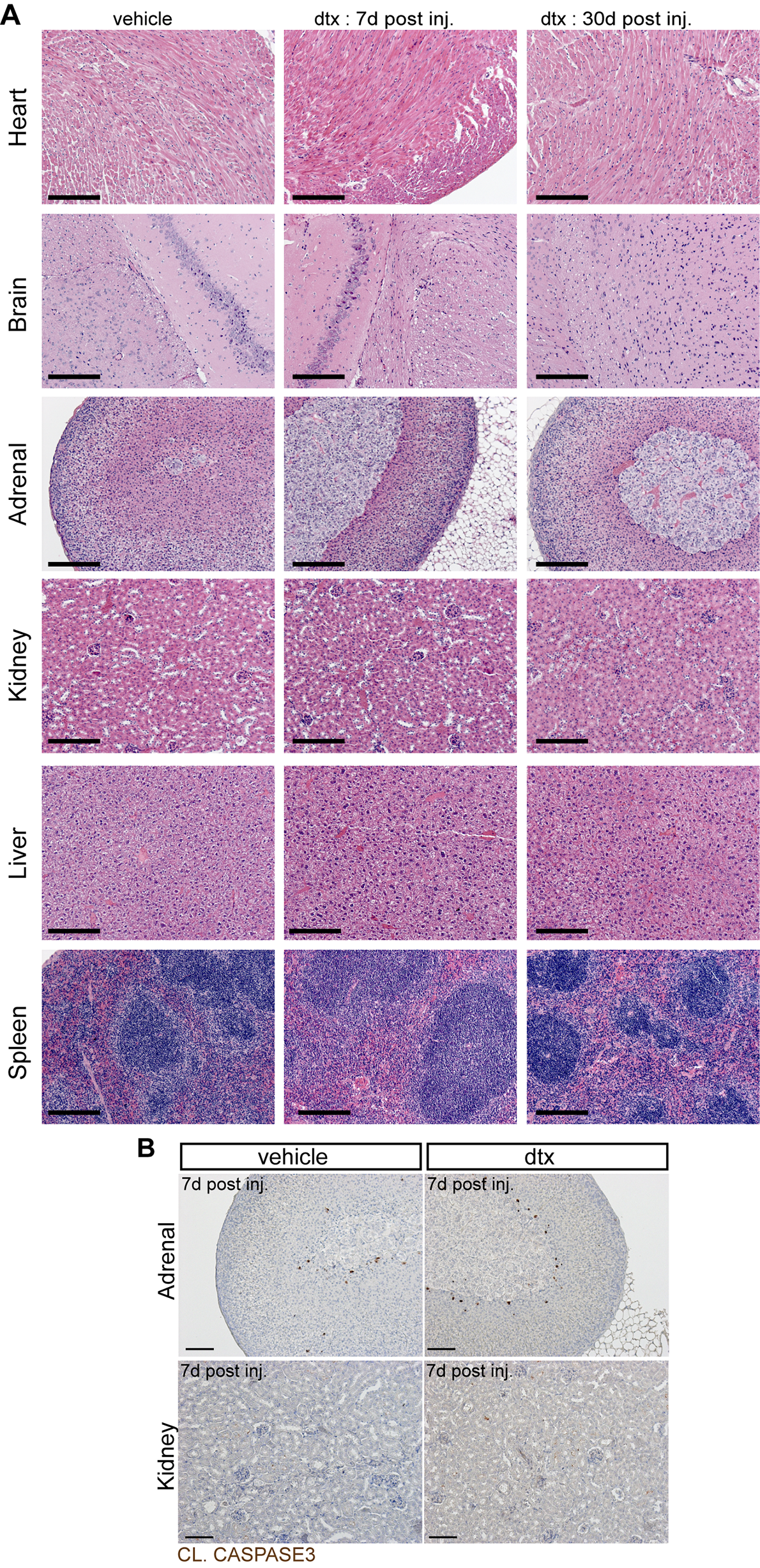

Supplement: Figure S2 — Diphtheria-toxin does not cause increased apoptosis in other organs. (A) Histology of non-gonadal tissues (Heart Brain Adrenal Kidney Liver and Spleen) demonstrating no off-target effects 7 or 30 days post-DTX injection (scale bar: 200 µm). (B) Immunostaining for CLEAVED CASPASE 3 protein in adrenal and kidney of adult Amh-Cre;iDTR vehicle or DTX-treated mice 7 days after ablation (scale bar: 100 µm). DTX treatment to ablate Sertoli cells engenders no off-target apoptosis in other tissues. d,days; dtx, injected with toxin; veh: vehicle control. (TIF) [file pone.0105687.s002.tif]
