# Supplementary material for: Sertoli Cells Maintain Leydig Cell Number and Peritubular Myoid Cell Activity in the Adult Mouse Testis
Source: PLoS One. 2014 Aug 21;9(8):e105687. doi: 10.1371/journal.pone.0105687 (PMC4140823; doi:10.1371/journal.pone.0105687)
Supplement: Table S1 — Primers used for Genotyping and qRT-PCR. (DOCX) [file pone.0105687.s003.docx]

**Table S1: Primers used for Genotyping and qRT-PCR**

| **Gene symbol** | **Primers 5’-3’** |  |
| --- | --- | --- |
| HBEGF (iDTR) mutant  HBEGF (iDTR) common  HBEGF (iDTR) wild type | CAT CAA GGA AAC CCT GGA CTA CTG |  |
|  | AAA GTC GCT CTG AGT TGT TAT |  |
|  | GGA GCG GGA GAA ATG GAT ATG |  |
| Stra8-Cre | GTG CAA GCT GAA CAA CAG GA |  |
|  | AGG GAC ACA GCA TTG GAG TC |  |
| GFP | GAC GTA AAC GGC CAC AAG TT |  |
|  | GGT CTT GTA GTT GCC GTC GT |  |
| Amh-Cre | CAC ATC AGG CCC AGC TCT AT |  |
|  | gtg tac agg atc ggc tct gc |  |
| *Fshr* | ggc cag gtc aac ata ccg ctt g |  |
|  | tgc ctt gaa ata gac ttg ttg caa att g |  |
| *Wt1* | gct cca gct cag tga aat gga cag aa |  |
|  | ggc cac tcc aga tac acg ccg |  |
| *Cnn1* | caa gct ggc cca gaa ata cga cc |  |
|  | tct tca cag aac ccg gct gca g |  |
| *Myh11* | ctg cac aac ctg agg gag cga tac t |  |
|  | aat ggc ata gat gtg agg cgg c |  |
| *Tnfa* | *CCC AGA CCC TCA CAC TCA GAT CATC* |  |
|  | *CCA CTA GTT GGT TGT CTT TGA GAT CCA TG* |  |
| *Cyp11a1* | cac aga cgc atc aag cag caa aa |  |
|  | gca ttg atg aac cgc tgg gc |  |
| *Stra8* | gaa ggt gcat ggt tca ccg tgg |  |
|  | gct cga tgg cgg gcc tgt g |  |
| *Dkkl1* | gag cca gaa cgg aac ccg ga |  |
|  | gac atc ggg ctg ggt cct cc |  |
| *Dhh* | AGA ACA GCG GCG CAG ACC G |  |
|  | GCC TTC GTA GTG GAG TGA ATC CTG TG |  |
| *Ptm2* | GGC GGC ATC GCA GAG GCT GC |  |
|  | CAC ATG ATG TTG CTT GGG CAG GT |  |
