# Supplementary material for: Sertoli Cells Maintain Leydig Cell Number and Peritubular Myoid Cell Activity in the Adult Mouse Testis
Source: PLoS One. 2014 Aug 21;9(8):e105687. doi: 10.1371/journal.pone.0105687 (PMC4140823; doi:10.1371/journal.pone.0105687)
Supplement: Table S2 — Details of Antibodies and detection methods used. (DOCX) [file pone.0105687.s004.docx]

**Table S2: Details of Antibodies and detection methods used**

| Primary Antibody (AbI) name | References | Lot number | Source | Citrate retrieval | Dilution  AbI | Secondary antibody (AbII) conjugated | Dilution  AbII | Detection  system |
| --- | --- | --- | --- | --- | --- | --- | --- | --- |
| CL.CASPASE 3 | Cell signalling (NEB) #9661 | #9661S | polyclonal | y | 1/100 | Biotin | 1/500 | DAB |
| SOX9 | Millipore  Ab5535 | 2383973 | polyclonal | y | 1/5000 | Peroxidase | 1/200 | IF |
| HSD3B | Santa Cruz Biotechnology  sc-30820 | L0313 | polyclonal | y | 1/750 | Peroxidase | 1/200 | DAB/IF |
| SMA | Sigma-Aldrich A2547 | 032M4822 | monoclonal | y | 1/5000 | Peroxidase | 1/200 | IF |
| DDX4 (MVH) | Abcam Ltd.  Ab13840 | GR149231-2 | polyclonal | y | 1/400 | Peroxidase | 1/200 | IF |
| CALPONIN | Abcam Ltd.  Ab46794 | GR77864-1 | monoclonal | y | 1/1000 | Peroxidase | 1/200 | IF |
| LAMININ | Abcam Ltd.  Ab11575 | 931348 | polyclonal | y | 1/1500 | Peroxidase | 1/200 | IF |
| MYH11 | Abcam Ltd.  ab53219 | GR53621-2 | polyclonal | y | 1/1000 | Peroxidase | 1/200 | IF |
| GFP | Molecular probes | 1024102 | polyclonal | y | 1/200 | Peroxidase | 1/200 | IF |
| DTR | Abcam Ltd.  ab66792 | GR19953-2 | monoclonal | n | 1/50 | Biotin | 1/500 | IF |

DAB: diaminobenzidine, IF: immunofluorescence, y:yes, n:no
